# A Comparative Study on the Efficacy, Safety and Cost Effectiveness of Gabapentin and Pregabalin in the Treatment of Neuropathic Pain

**DOI:** 10.7759/cureus.95916

**Published:** 2025-11-01

**Authors:** Riya Kataria, Kranthi K Kadal, Sundar Shanmugam, Pallavi Setya

**Affiliations:** 1 Internal Medicine, Ashford and St. Peter's Hospital, Chertsey, GBR; 2 Pharmacology, Bhaarath Medical College and Hospital, Chennai, IND; 3 Neurology, Sri Ramachandra Institute of Higher Education and Research, Chennai, IND; 4 Internal Medicine, Lincoln County Hospital, Lincoln, GBR

**Keywords:** gabapentin, mcgill questionnaire, neuralgia, neuropathic pain, pregabalin, vas score

## Abstract

Introduction

Neuropathic pain distinguishes itself from nociceptive pain not only in its etiology and clinical presentation but also in its limited responsiveness to non-steroidal anti-inflammatory drugs (NSAIDs) and opioid analgesics. Gabapentin and pregabalin, collectively referred to as gabapentinoids, are anticonvulsant agents that have demonstrated efficacy in managing neuropathic pain. Evidence supports their use in comparison to placebo or antidepressants, such as tricyclic antidepressants, for the treatment of neuropathic pain. However, despite extensive investigation, there remains a paucity of conclusive data and often contradictory findings regarding the relative superiority of one drug over the other, particularly in resource-constrained settings and including the practicality of the money spent.

Aim

The aim of the study is to evaluate and compare the efficacy, safety parameters and cost-effectiveness of gabapentin (GB) and pregabalin (PG) in neuropathic pain.

Methods

A randomized double-blind comparative study was conducted to evaluate the effectiveness of GB and PG in the treatment of neuropathic pain, involving 60 patients attending the Neurology outpatient department (OPD) at Sri Ramachandra Institute of Higher Education and Research, Chennai. The participants were equally divided into two groups: Group A received GB and Group B received PG. Both groups were followed up regularly at four, eight, and 12 weeks. From day 0, doses were titrated according to the response and side effects, costs were noted down at each visit and pain was assessed using the Visual Analog Scale (VAS) and the McGill Pain Questionnaire.

Results

Both GB and PG demonstrate significant effectiveness in improving the symptoms of the patients after three months of treatment (2.5±0.9 for PG vs. 4.5±1.3 for GB, p<0.001) according to VAS, with a similar result when analysed with the McGill Pain Questionnaire. PG also has the advantages in terms of statistical results and evidence along with fewer reported adverse effects and better patient compliance.Cost evaluation indicated that the cumulative expense for PG over three months was INR 1,286 compared to INR 3,420 for GB, even with dose adjustments. Moreover, PG demonstrated a 75% reduction in cost per VAS point and a 72% decrease in cost per McGill point, highlighting its greater cost-effectiveness along with improved clinical results.

Conclusion

This randomized prospective study provides a comparison of the efficacy and safety of GB and PG in neuropathic pain, while throwing light into health economic part by including cost analysis. Such comparisons, including expense analysis, are limited in the literature. Our study plays a role in substantiating PG as a better analgesic in many respects for neuropathic pain while also proposing a possibility for evaluating combination therapies and to provide an optimize healthcare resource allocation.

## Introduction

Neuropathic pain is the pain initiated or caused by a primary lesion or dysfunction of the nervous system. It is often characterized by sensations such as burning, gnawing, aching, shooting, or lancinating pain. Although nerve damage can arise from various causes, the presence or severity of pathology does not always correlate with the intensity of the pain experienced [1].

Neuropathic pain is broadly classified based on disorders of the peripheral or central nervous systems. The common causes of peripheral neuropathic pain include diabetes mellitus, porphyria, herpes zoster infection, HIV-related neuropathies, nutritional deficiencies, drug-induced neuropathies (e.g., paclitaxel, vinca alkaloids), uremia, chronic liver diseases, genetic or immune-mediated disorders, and physical trauma to nerve trunks. Conversely, central neuropathic pain typically results from spinal cord injury or stroke [2].

Extensive evidence supports the use of antidepressants (tricyclic antidepressants and serotonin-norepinephrine reuptake inhibitors) and anticonvulsants (gabapentin and pregabalin) as first-line treatments for neuropathic pain [3]. GB (gabapentin) and PG (pregabalin) belong to a unique class of compounds characterized by their high-affinity binding to the α2δ protein, an auxiliary subunit of voltage-gated calcium channels in the central nervous system tissues. This binding modulates the release of neurotransmitters [1]. GB was initially approved for the treatment of epilepsy (partial seizures) but soon demonstrated efficacy in managing neuropathic pain syndromes, such as postherpetic neuralgia [4]. In 2005, PG became the first medication approved for managing neuropathic pain associated with diabetic peripheral neuropathy and postherpetic neuralgia in adults, as well as an adjunctive therapy for partial-onset seizures in adults [5].

Furthermore, in developing nations such as India, it has been noted that PG is known to show a greater cost-effectiveness than GB, primarily due to its superior and more stable bioavailability and linear pharmacokinetics, unlike GB, whose absolute bioavailability declines with higher doses. Both GB and PG have demonstrated efficacy as therapeutic agents for neuropathic pain in both preclinical and clinical studies. While both drugs are widely used to treat neuropathic pain, there remains a lack of consensus regarding their comparative effectiveness [4]. 

The study aims to compare the therapeutic performance of GB and PG in managing neuropathic pain, focusing on their therapeutic outcomes and effectiveness, including the costs of healthcare resources and the health outcomes achieved by treatment. Additionally, it seeks to evaluate the pharmacoeconomic efficacy of these drugs through a cost-utility analysis, providing insights into their economic viability and overall value in clinical practice. This dual approach aims to guide clinicians in making professional decisions about optimal treatment strategies for neuropathic pain.

## Materials and methods

Ethical clearance

The study protocol was granted approval by the Institutional Ethics Committee at Sri Ramachandra Institute of Higher Education and Research (deemed to be university), Chennai, India (Approval No: CSP/21/AUG/97/405). All participants provided written informed consent after receiving detailed information - either in English or the local language (Tamil) - regarding the study's objectives, procedures, and potential risks.

Study design and setting

This is a randomized double-blind comparative study conducted at Sri Ramachandra Institute of Higher Education and Research, Chennai, India, over a three-month period from July 2022 to September 2022.

Sample size calculation

The sample size for the above study is based on a published report by Amit Kumar Ghosh et al., which mentioned the mean change in Pain Quality Assessment Scale (PQAS) score after gabapentin therapy was noted as 31.32, and after pregabalin therapy as 40.56, with an average standard deviation of 15 in both groups [6]. The following formula was used to calculate the sample size:

$N = \frac{(Z_{1-\alpha/2} + Z_{1-\beta})^2 \cdot 2\sigma^2}{(\mu_1 - \mu_2)^2}$

where

$Z_{1-\alpha/2} = 1.96$ (95% confidence interval)

$Z_{1-\beta} = 0.84$ (80% power)

$\mu_1 = 31.32$ (Gabapentin group)

$\mu_2 = 40.56$ (Pregabalin group)

$\sigma=\sigma_{15}=15$ (average SD)

The final calculated sample size was 41.37 per group, which after adjustment for a 10% non-response rate yielded a required sample size of 45 per group (90 total).

However, due to time constraints, only 60 patients (30 per group) were officially recruited for this study.

Participants

Inclusion Criteria

Patients aged 18 years or older with a clinical diagnosis of neuropathic pain were eligible, including conditions such as diabetes mellitus, fibromyalgia, and post-herpetic neuralgia were included in the study.

Exclusion Criteria

Patients with pregnancy, psychiatric illness, spinal cord injury, or any complex regional pain syndrome were excluded.

Randomization and allocation

The 60 officially enrolled patients were randomized into two equal groups: Group A (gabapentin, n=30) and Group B (pregabalin, n=30). Eligible participants were assigned in a 1:1 ratio to either gabapentin or pregabalin using a computer-generated random-number sequence. Sequential assignment maintained allocation concealment and minimized selection bias. 

Variables

The primary outcomes were recorded as pain intensity and symptom relief, measured using the Visual Analogue Scale (VAS) and McGill Pain Questionnaire at baseline (day 0), one month (day 30), two months (day 60), and study completion (day 90). Secondary outcomes included tolerability (adverse events) and more importantly the cost-effectiveness (direct medication costs per unit reduction in VAS/McGill scores).

Data sources and measurement

At each follow-up, patient history was reviewed, and reassessments were conducted using VAS and McGill Pain Questionnaire, along with recording of any adverse effects at different dosages. Compliance with medications was monitored using the pill-count method.

Interventions and dosing adjustments

Gabapentin (Group A): 100 mg thrice daily (300 mg/day) initially → escalated to 300 mg twice daily (600 mg/day) at one month. By two months, 30% remained on 600 mg/day, while 70% required escalation to 900 mg/day.

Pregabalin (Group B): 75 mg once daily initially → escalated to 75 mg twice daily (150 mg/day) at one month. By two months, 82% continued at this dose, while 18% required escalation to 300 mg/day.

Adherence and adverse event monitoring

Medication adherence was verified through pill-count checks at each follow-up. Adverse drug reactions (ADRs) were recorded systematically using a structured data sheet, and severity was graded according to standard pharmacovigilance criteria. Participants showing <80 % adherence were flagged for sensitivity review.

Statistical analysis

The collected data were entered into Microsoft Excel (Microsoft Corp., Redmond, WA) and analysed using IBM SPSS Statistics, version 20 (IBM Corp., Armonk, NY, USA). Descriptive statistics were presented as means with standard deviations (SD) for continuous variables and as frequencies with percentages for categorical variables. The distribution of demographic characteristics (age and sex) between the two groups was then assessed using the independent t-test and chi-square test, respectively.

The comparisons of mean pain scores between the groups at baseline and subsequent follow-up intervals (one, two, and three months) were performed using the independent t-test. Within-group changes over time were evaluated using repeated measures such as analysis of variance (ANOVA). The VAS and McGill Pain Questionnaire scores were similarly analyzed across time points, and significance was determined by independent t-tests at each interval.

The categorical distribution of pain descriptors (distress, discomfort, mild pain, no pain) was contrasted between the groups using the chi-square test. Proportions of patients achieving pain-free status at three months were also equated using the chi-square test. No post-hoc subgrouping was performed.

A p-value of <0.05 was considered statistically significant for all analyses, whereas values below 0.01 and 0.001 were regarded as highly significant.

Pharmacoeconomic evaluation (cost-effectiveness)

The pharmacoeconomic evaluation was performed from the viewpoint of the patient, concentrating on the direct costs of medications during the three-month treatment period. Drug-price data were obtained from the average retail prices of commonly used generic brands in Chennai (2022), verified with the Current Index of Medical Specialities (CIMS India) and the 1mg online drug database. Cumulative monthly expenses were computed according to dose-escalation trends, and incremental cost-effectiveness ratios (ICERs) were calculated based on cost per unit reduction in VAS and McGill Pain Questionnaire scores.

Indirect costs - such as lost productivity, travel, and caregiver time - and intangible costs, including pain-related quality-of-life decrements, were excluded since the study’s goal was to evaluate short-term clinical pharmacoeconomics. Moreover, all our patients were recruited from the outpatient department and none of them was affected by any financial difficulties due to illness. It is to be noted that most of the patients were from the same locality, rendering any travel costs as nil. Future prospective studies with extended follow-up may incorporate these aspects for a broader societal-perspective analysis.

## Results

The study included 30 patients in each group. The mean age was 45.2 years for Group A (GB) and 46.8 years for Group B (PG). Group A had equal male and female patients (15 each), while Group B had 18 men and 12 women.

The two groups were assessed over a period of three months using VAS and McGill Pain Questionnaire. At baseline, the VAS scores were comparable between Group A (GB: 8.5±1.0) and Group B (PG: 8.6±1.1), with minimal statistical difference (p=0.76) as demonstrated in Table 1. At the end of the first month, Group B showed a higher reduction in pain (5.0±1.0) as compared to Group A (6.5±1.1), which was statistically significant (p<0.05). This difference broadened even further at two months (3.5±0.8 for PG vs. 5.2±1.0 for GB, p<0.01) and then finally at the end of the study, the results clearly showed effective pain reduction in Group B (2.5±0.9 for PG vs. 4.5±1.3 for GB, p<0.001).

**Table 1:** VAS score results for a period of three months.

Assessment Time	Group A: Gabapentin (Mean±SD)	Group B: Pregabalin (Mean±SD)	p-value
Pre-treatment	8.5±1.0	8.6±1.1	0.76
1 Month	6.5±1.1	5.0±1.0	<0.05 (0.032)
2 Months	5.2±1.0	3.5±0.8	<0.01 (0.008)
3 Months	4.5±1.3	2.5±0.9	<0.001 (0.001)

**Figure 1:**
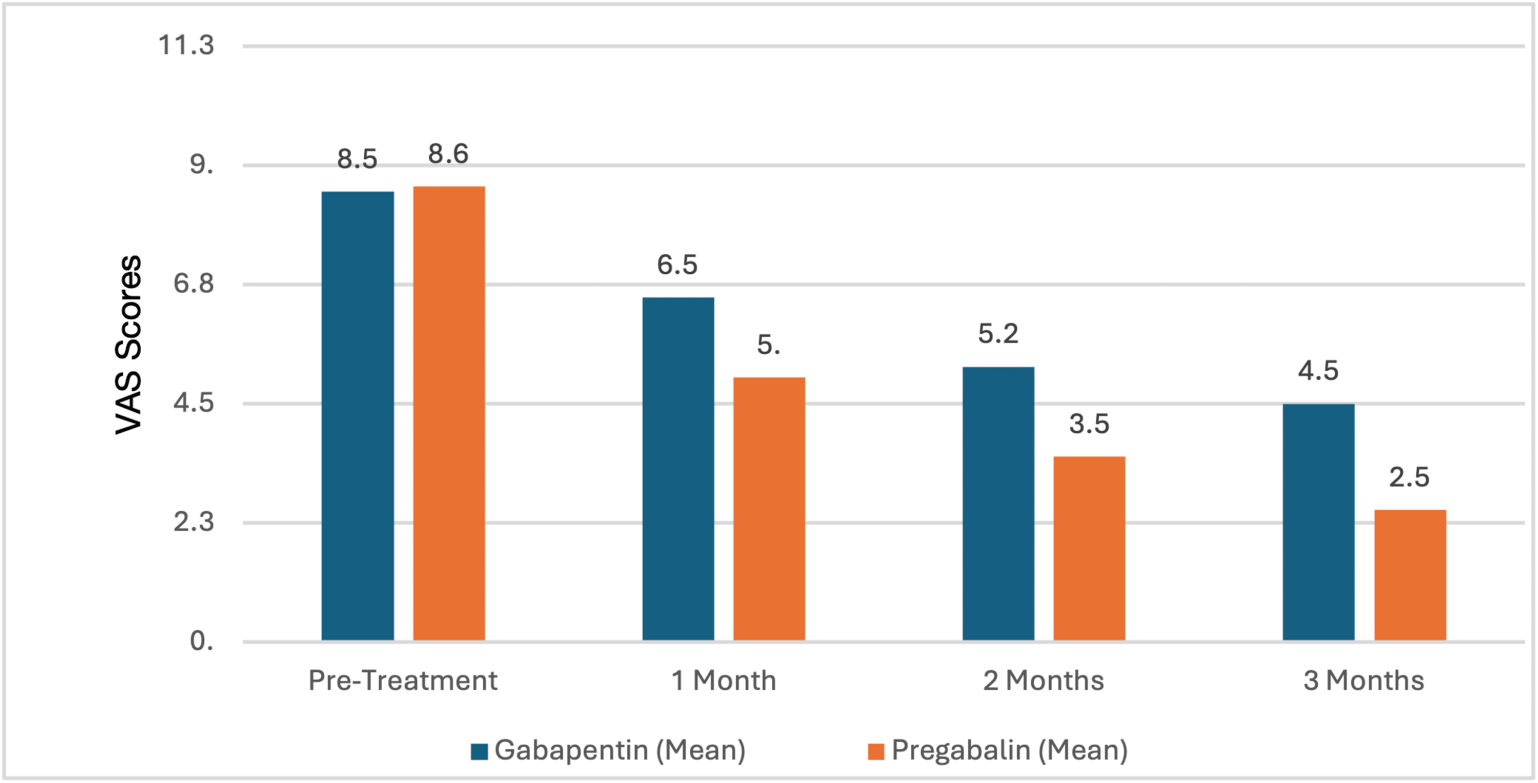
Comparing the VAS score results for GB and PG groups for a period of three months

These results definitely suggest that while both the above-mentioned drugs, GB and PG, potently helped in the reduction of neuropathic pain, PG consistently provides significantly greater pain reduction across all follow-up periods (Figure 1).

**Figure 2:**
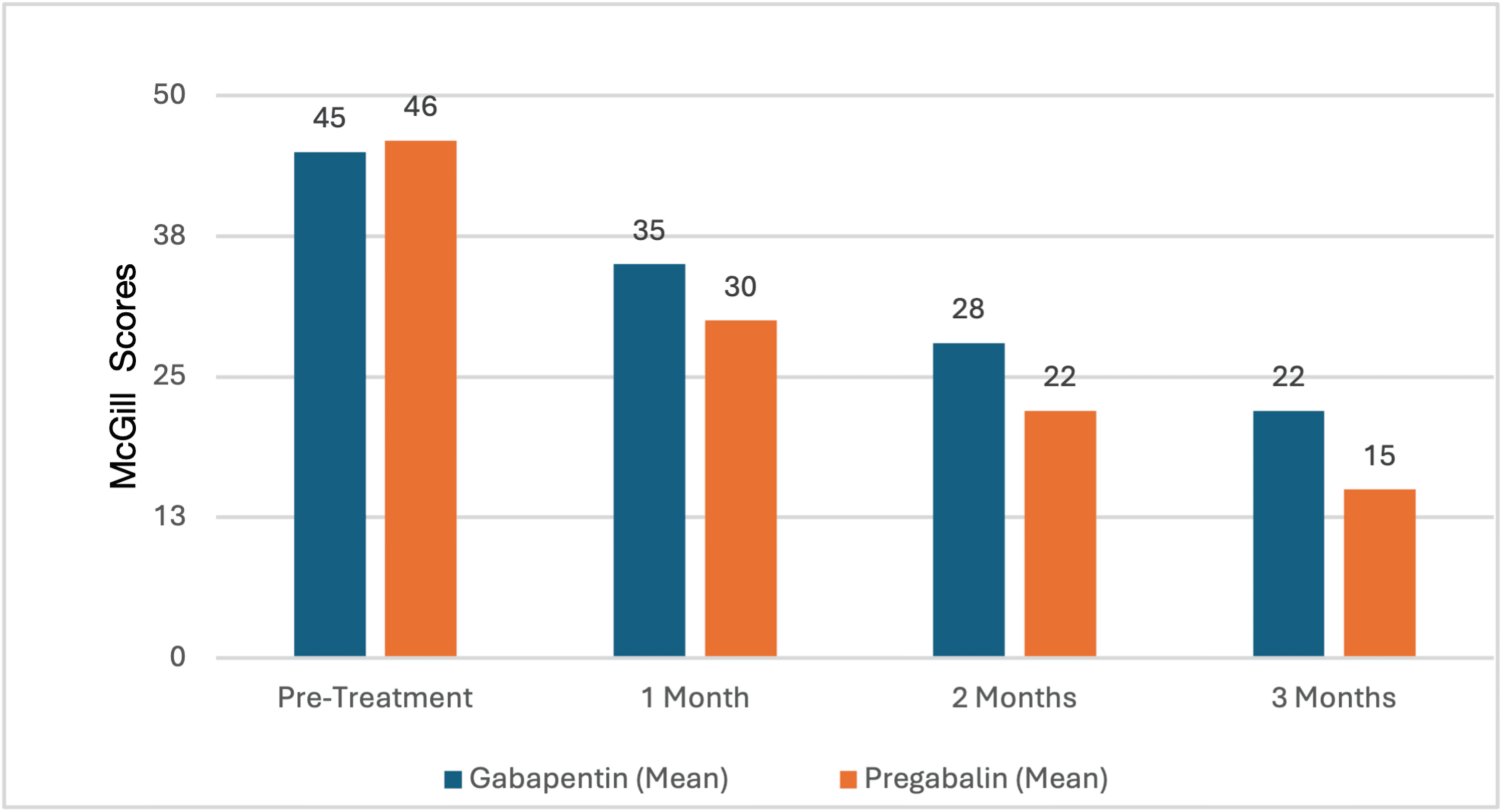
Comparing McGill score results for the two groups over a period of three months.

The McGill Pain Questionnaire was then used to assess the pain intensity in the two groups periodically over three months (Table 2). At the initial pre-treatment assessment, the pain scores were noted to be similar between patients on GB (45±10) and on PG (46±11), with no significant difference (p=0.78). At the first month follow-up, Group B patients demonstrated a greater pain reduction (30±7) as compared to the other group (35±8) with a p-value <0.05. This trend continued at two months as well, where a mean score of 22±6 was noted for Group B and 28±7 for Group A (p<0.01). At three months, Group B (15±6) finally outperformed Group A (22±5) in its efficacy to reduce pain with a highly significant difference (p<0.001), as also shown in Figure 2.

**Table 2:** McGill Pain Questionnaire results for gabapentin and pregabalin

Assessment Time	Group A: Gabapentin (Mean ± SD)	Group B: Pregabalin (Mean ± SD)	p-value
Pre-treatment	45±10	46±11	0.78
1 Month	35±8	30±7	<0.05 (0.032)
2 Months	28±7	22±6	<0.01 (0.008)
3 Months	22±5	15±6	<0.001 (0.001)

In addition to the above results, the VAS scores also revealed marked dissimilarities between the two groups at each assessment points in terms of pain description (Table 3). At the first follow-up assessment, 70% of Group A patients reported distress, whereas Group B patients predominantly complained of discomfort (82%) and mild pain (18%) (p<0.001). By two months, Group A patients largely reported discomfort (60%) and mild pain (32%), while Group B patients exhibited a greater proportion of mild pain (77%) and a considerably lower incidence of discomfort (23%) (p<0.001).

**Table 3:** Comparing pain category outcomes for the two groups using Visual Analog Score.

Time period	Pain category	Visual Analog Score (VAS)		p-value
		Gabapentin (%)	Pregabalin (%)	
After 1 month	Distress	70	-	0.001
	Discomfort	22	82	0.001
	Mild	8	18	0.001
After 2 months	Distress	8	-	0.001
	Discomfort	60	23	0.001
	Mild	32	77	0.001
After 3 months	Discomfort	12	3	0.001
	Mild	88	97	0.001

A similar trend was recorded on comparison of the McGill pain scores which yet again highlighted PG’s better pain relief quality at all time periods (Table 4).

**Table 4:** Comparing pain category outcomes using McGill Pain Questionnaire

Time period	Pain category	McGill Pain Scores		p- value
		Gabapentin (%)	Pregabalin (%)	
After 1 month	Moderate	80	20	<0.001
	Mild	10	80	0.002
After 2 months	Moderate	30	18	0.001
	Mild	40	-	0.001
	No pain	5	40	0.001
	Mild	25	42	0.001
After 3 months	No pain	45	67	0.001
	Mild	40	33	0.001
	Moderate	15	-	0.001

The next aspect of the study consisted of analyzing the cost effectiveness of the drugs with respect to the low economic population. A single GB 100 mg strength standard tablet costs around INR 6.67, while a single 300 mg GB tablet costs INR 20. It was noted that the individual cost of a PG 75 mg tablet was INR 8. As shown in Table 5, PG demonstrated a distinct pharmacoeconomic benefit, with a total expense of INR 1,286 over three months compared to INR 3,420 for GB, even after dose adjustments. Although 18% of patients on PG needed to increase their dosage to 150 mg twice a day by the third month, the overall financial impact was modest due to the drug's consistent cost per milligram. In contrast, GB's expenses escalated significantly as 70% of patients required a higher dosage of 300 mg three times daily (900 mg/day), which considerably raised monthly costs. This difference in expenditure highlights PG's more predictable and cost-effective profile, positioning it as a more practical choice in healthcare environments with limited resources.

**Table 5:** Month-wise cost comparison (INR) of GB and PG with dose adjustment GB: Gabapentin; PG: Pregabalin.

Month	Regimen	Group A: Gabapentin (GB)	Group B: Pregabalin (PG)
1	Starting Dose	100 mg TID (INR 20/day)	75 mg OD (INR 8/day)
	Monthly Cost	INR 600 (INR 20 × 30 days)	INR 240 (INR 8 × 30 days)
	Cumulative Cost	INR 600	INR 240
2	Dose Adjustment	300 mg BID (INR 40/day)	75 mg BID (INR 16/day)
	Monthly Cost	INR 1,200 (INR 40 × 30 days)	INR 480 (INR 16 × 30 days)
	Cumulative Cost	₹1,800 (₹600 + ₹1,200)	INR 720 (INR 240 + INR 480)
3	Dose Adjustment	- 30% of patients on 300 mg BID (₹40/day)	- 82% of patients on 75 mg BID (₹16/day)
		- 70% of patients on 300 mg TID (₹60/day)	- 18% of patients on 150 mg BID (₹32/day)
	Monthly Cost (Weighted Average)	(0.30×INR 1,200) + (0.70×INR 1,800) = ₹1,620	(0.82×INR 480) + (0.18×INR 960) = INR 566 (approx.)
	Cumulative Cost	INR 3,420 (INR 1,800 + INR 1,620)	INR 1,286 (INR 720 + INR 566)

PG further demonstrated significantly better cost-effectiveness, with costs per VAS point being 75% lower (INR 211 compared to INR 855) and costs per McGill point being 72% lower (INR 41.5 compared to INR 149) in comparison to GB. The higher clinical benefits and reduced incremental cost associated with PG highlight its importance for long-term management of neuropathic pain in resource-limited environments.

From a cost-effectiveness standpoint, PG (75 mg twice daily or 150 mg twice daily) is the more economical option, costing not more than INR 32 per day (for the 150 mg BD subgroup). However, GB (300 mg thrice daily) costs INR 60 per day, indicating that PG offers comparable and potentially superior outcomes at a lower cost (Table 6).

**Table 6:** Cost-effectiveness comparison over the course of the study: three-month period

Parameter	Group A: Gabapentin (GB)	Group B: Pregabalin (PG)	Difference
Total cost (INR)	INR 3,420	INR 1,286	INR 2,134 saved
VAS improvement (8.6 → 2.5)	4.0 points	6.1 points	+2.1 points
Cost/VAS point (INR/point)	INR 855	INR 211	75% lower with PG
McGill improvement (46 → 15)	23 points	31 points	+8 points
Cost/McGill point (INR/point)	INR 149	INR 41.5	72% lower with PG

Moreover, both the drugs, GB and PG, exhibited analogous safety profiles in the above study, with mild drowsiness and occasional headaches being the only reported adverse effect in a few patients. 

## Discussion

In our study, the results from both the scoring systems clearly indicate that though both the above-mentioned drugs are effective in alleviating neuropathic pain. PG offers superior pain relief at every assessment point, further reinforcing its potential as a better option for managing chronic pain. At the end of the study, PG’s superior efficacy in alleviating both distress and discomfort was established as 97% of Group B patients reported mild pain as compared to 88% of Group A patients. Patients on PG also demonstrated a substantially lower rate of discomfort (3% vs. 12%) (p=0.001). 

Neuropathic pain can be difficult to diagnose and is often missed in primary care setups [7,8]. Nerve conduction studies and somatosensory evoked potentials can help in confirming the diagnosis of neuropathy, but they tend to measure function in only large myelinated fibres [9]. Quantitative sensory testing is another sophisticated neurophysiologic technique to test for abnormally increased function or any loss of function in both large and small nerve fibres [10]. Furthermore, simple questionnaires such as the Leeds Assessment of Neuropathic Symptoms and Signs (LANSS) and Douleur Neuropathique en 4 Questions (DN4), also help in differentiating between somatic pain and neuropathic pain [11,12]. In our study, the VAS and McGill Pain Questionnaire were used as the evidence supports the validity of these questionnaires for the assessment of pain intensity and evaluating periodic therapeutic response [13,14].

The first line of treatment for neuropathic pain is antidepressants (Amitriptyline), PG and GB, as used in the current study. If initial treatment is rendered ineffective, lidocaine, botulinum toxin and potent opioids are considered as second or third line options [15]. 

In the literature, the three common drugs - Amitriptyline, PG and GB - have been extensively discussed, however, only four studies have compared the treatment effects of GB and PG in a head-to-head manner for neuropathic pain [16,17]. Mishra et al. reported significantly increased pain reduction with PG 600 mg daily as compared to GB 1800 mg daily with p=0.042 [18]. This is directly comparable to our results, where, after three months of treating the patients with varying doses of GB and PG, as many as 67% patients on PG reported no pain; however, only 45% patients in Group A reported the same. Contrarily, another study reported GB 2400 mg daily to be more effective in reducing pain intensity than PG 600 mg daily (p=0.035), with added adverse events associated with PG than GB (p=0.002) [19]. Rauck et al. further compared the two drugs with no significant differences in efficacy [20]. 

Ghosh et al. proposed that PG may provide faster pain relief compared to GB, which could be attributed to its stronger binding affinity for the α2-δ subunit of voltage-gated calcium channels and its predictable, linear pharmacokinetics after oral intake [6]. Other studies have also noted several possible advantages of PG over GB in neuropathic pain therapy. While both of these newer anticonvulsants generally have limited drug interactions, one key issue with GB is the challenge of achieving optimal dosing. This can result in insufficient pain relief and the mistaken perception that the drug is ineffective [21-23]. Moreover, GB’s nonlinear pharmacokinetic pattern may complicate dose adjustments, making it harder to reach effective levels while keeping side effects under control [7]. In the above study, majority of the patients in Group A required a dose escalation to 900 mg daily (300 mg thrice daily) to address the pain experienced by the patients. As also noted in a recent review, it has been suggested that PG is more effective in reducing pain compared to GB in short‐term follow‐up (six weeks or less), but there was no difference between the two drugs in long‐term follow‐up (six weeks or more) [24]. It is important that the above-mentioned points are also considered by the physicians while prescribing these drugs to patients with long-term neuropathic pain.

Safety and tolerability can be a matter of great importance while managing chronic diseases such as neuropathic pain as such patients have an already excessively affected health status. As reported in previous studies, GB generally demonstrates minimal side effects and non-organ toxicity [25]. PG has a better bioavailability profile and its minimal drug interactions with lack of interference with hepatic enzymes gives it an advantage over GB [26]. Specifically, the most commonly seen adverse effects of the two drugs are dizziness and somnolence (dose-related), and these two symptoms also present during withdrawals [27]. As also demonstrated in our study, the side effects are usually mild to moderate in severity and are tolerated well if compared to other treatment options like tricyclic antidepressants, opioids and non-steroidal anti-inflammatory drugs [28].

Moving on to the cost analysis of the drugs, our evaluation revealed that PG clearly incurred a lower total expense over a three-month period (INR 1,286 compared to INR 3,420 for GB), while also achieving better clinical results in pain assessments. Bockbrader et al. substantiated that PG has superior and more stable bioavailability than GB, reinforcing its effectiveness at reduced dosages [4]. Cost-effectiveness evaluations from various healthcare contexts, including Canada and Greece, have consistently indicated that PG results in better clinical outcomes at a lower or comparable cost overall [29,30]. These results collectively showcase PG's role as an effective and economically smart first-line treatment for neuropathic pain in countries like India with a huge population and somewhat limited resources.

In the patient population included in our study, PG provided an increased and better pain reduction. It was noted that though both the drugs have potent analgesic properties for neuropathic pain, PG proved to have a significant effect on quickly alleviating symptoms while being cost-effective for patients.

Limitations

This study has several constraints that should be acknowledged. The most notable are the relatively small sample size. This smaller sample size may have reduced the statistical power of our comparisons, thereby limiting the generalizability of the findings. Second, the short duration of follow-up (three months) may not fully capture the long-term efficacy and safety of gabapentin and pregabalin in chronic neuropathic pain management. In addition, the absence of a proper placebo control group limited the strength of comparisons between treatment arms. The study was approved by the institutional ethics committee (IEC); however, it was not registered with a trial registry. It is also possible that participants in both groups held expectations or preconceived notions about pain relief, which could have affected their reporting. Conversely, some individuals may have had negative attitudes toward the prolonged use of a specific drug, particularly since they were not permitted to switch to the alternate treatment. Although all patients were counseled regarding supplementary non-pharmacological measures for neuropathic pain, the study design did not allow strict monitoring or regulation of these practices, nor of the use of over-the-counter medications. In summary, the findings of this work cannot be equated with the results of a large, randomized, double-blind, placebo-controlled clinical trial directly comparing the two drugs.

Strengths

Despite these limitations, our study has distinguished strengths. It was designed prospectively, allowing for systematic follow-up and consistent assessment of outcomes over a three-month period. Standardized, validated and easy-to-use tools (VAS and McGill Pain Questionnaire) were employed to evaluate pain, ensuring reliability and comparability of results. The study also incorporated a stepwise dosing strategy reflecting real-world clinical practice, thereby enhancing external validity. Furthermore, by including a cost-effectiveness analysis alongside clinical outcomes, the study hence provides a pragmatic perspective on the economic implications of GB and PG use, which is of particular relevance in resource-limited settings.

## Conclusions

Both gabapentin and pregabalin proved effective in alleviating neuropathic pain; however, pregabalin demonstrated greater efficacy, tolerability and cost-efficiency. The interpretation of results should consider the study limitations, notably the lack of blinding and exclusion of indirect cost components. Future large-scale, long-term analyses are recommended to confirm these findings across diverse healthcare environments.

## Appendices

**Table 7:** Short Form McGill Pain Questionnaire [14,15]

	None	Mild	Moderate	Severe
Throbbing	0	1	2	3
Shooting	0	1	2	3
Stabbing	0	1	2	3
Sharp	0	1	2	3
Cramping	0	1	2	3
Gnawing	0	1	2	3
Hot/Burning	0	1	2	3
Aching	0	1	2	3
Heavy	0	1	2	3
Tender	0	1	2	3
Splitting	0	1	2	3
Tiring/Exhausting	0	1	2	3
Sickening	0	1	2	3
Fearful	0	1	2	3
Punishing/Cruel	0	1	2	3
